# Chest Wall to Heart Distance Reproducibility in Postoperative Deep Inspiration Breath-Hold Radiotherapy for Left-Sided Breast Cancer Using an Anzai Laser Sensor With Visual Feedback

**DOI:** 10.7759/cureus.53183

**Published:** 2024-01-29

**Authors:** Masataka Hoshina, Masaya Noguchi, Hirotoshi Sekihara, Koichi Masuda, Mitsuko Shinmura, Shinji Sugahara

**Affiliations:** 1 Radiology and Radiation Oncology, Tokyo Medical University Ibaraki Medical Center, Inashiki-gun, JPN; 2 Nursing, Tokyo Medical University Ibaraki Medical Center, Inashiki-gun, JPN

**Keywords:** dibh, heart v25gy, mean heart dose, radiotherapy, breath-hold, visual feedback, laser sensor, distance reproducibility, chest wall to heart, left-sided breast cancer

## Abstract

Background

Left-sided breast cancer radiotherapy may increase the risk of cardiovascular death due to possible heart irradiation. The reproducibility of the chest wall to heart distance in deep inspiration breath-hold (DIBH) was studied using a laser sensor with visual feedback.

Methodology

A total of 10 consecutive postoperative left-sided breast cancer cases receiving DIBH radiotherapy between December 2022 and September 2023 were retrospectively investigated. The prescribed dose was 50 Gy in 25 fractions. An Anzai respiratory gating system, AZ-733VI (Anzai, Tokyo, Japan), was employed that has a laser displacement sensor and a visual feedback device. An Elekta linac with a cone-beam CT unit, Axesse (Elekta AB, Stockholm, Sweden), was used in this study. The interfractional changes in the chest wall to heart distance among 25 fractions were analyzed for each of the 10 patients in each coordinate axis. In addition, the median with the 95% confidence interval (CI) and interquartile range (IQR) for all 250 fractions were calculated in each axis to assess the reproducibility of our DIBH technique.

Results

The medians of the interfractional changes in the chest wall to heart distance in each of the 10 patients ranged from -2 mm to 3 mm, -1 mm to 3 mm, and -2 mm to 1 mm in the lateral (X), superior-inferior (Y), and anterior-posterior (Z) directions, respectively. For all 10 cases, the medians were 1 mm (95% CI = 0.72 to 1.28 mm) in X, 1 mm (95% CI = 0.76 to 1.24 mm) in Y, and 0 mm (95% CI = -0.20 to 0.20 mm) in Z directions, whereas the IQRs were 4 mm in X, 2 mm in Y and 2 mm in Z directions. The measured IQRs were two to three times smaller than those shown in a previous report without visual feedback, suggesting a clinical advantage of the visual feedback in DIBH for left-sided breast cancer radiotherapy. The DIBH solution shown in this study required approximately 10 minutes from room-in to room-out, thereby not reducing the daily number of patients.

Conclusions

Our DIBH approach with visual feedback achieved better distance reproducibility between the chest wall and heart by a factor of two to three in terms of IQR compared to the previously reported data without visual feedback. Patient throughput was also favorable. To our knowledge, this is the first report demonstrating the chest wall to heart distance reproducibility in DIBH with visual feedback.

## Introduction

Left-sided breast cancer radiotherapy may increase the risk of cardiovascular death due to possible heart irradiation. The Quantitative Analysis of Normal Tissue Effects in the Clinic guideline stated that heart V25Gy <10% would lead to long-term cardiac mortality of below 1% based on published normal tissue complication probability (NTCP) models [1]. Moiseenko et al. reported that heart V25Gy of 5% would correspond to cardiac mortality of 1% by referring to an NTCP model with parameters estimated from their data [2]. Beaton et al. stated that the risk of radiation-induced cardiac death at 10 years appeared to be very low if the mean heart dose (MHD) and the heart V25Gy were below 3.3 Gy and 5%, respectively, based on their analysis including 2,644 left-sided breast cancer patients [3].

Sung et al. showed that deep inspiration breath-hold (DIBH) resulted in much lower MHD and heart V25Gy compared to free breathing (FB) by referring to their planning results of 22 patients [4]. Voluntary DIBH (vDIBH) was extensively studied by Bartlett et al. with good target reproducibility using a light field edge as a marker [5-7]. Yamauchi et al. reported that DIBH with visual feedback reduced the mean chest wall displacements from 2.1 mm to 0.6 mm in a study involving 43 patients [8]. Van Haaren et al. measured daily changes in the distance from the heart to the chest wall relative to the plans for 50 patients receiving vDIBH radiotherapy without visual feedback, showing a maximum distance reduction of 8.3 mm relative to the plan [9]. Benkhaled et al. also reported the distance reproducibility for 15 patients receiving vDIBH without visual feedback, where cone-beam CT (CBCT)-based image registration was retrospectively performed twice, one for the chest wall and the other for the heart [10]. They calculated daily changes in the chest wall to heart distance relative to each plan by subtracting registration errors for the heart from those for the chest wall. They showed the largest distance reductions of 21 mm toward the left, 9 mm toward the superior, and 18 mm toward the anterior directions. They also reported that the interquartile ranges (IQRs) were 8.0 mm in the lateral direction, 4.8 mm in the superior-inferior direction, and 6.4 mm in the anterior-posterior direction. An European Society for Radiotherapy and Oncology-Advisory Committee for Radiation Oncology Practice guideline for breath-hold radiotherapy was also published for various tumor sites including breast cancers [11].

According to our DIBH literature review, the chest wall to heart distance reproducibility with visual feedback has not been reported. Therefore, we have investigated the distance reproducibility using our DIBH solution with visual feedback. We hypothesized that the visual feedback may increase the distance reproducibility. To our knowledge, this is the first paper reporting the chest wall to heart distance reproducibility with visual feedback.

## Materials and methods

In this study, 10 consecutive postoperative left-sided breast cancer cases receiving DIBH radiotherapy between December 2022 and September 2023 were retrospectively investigated. The patients’ demographics are shown in Table 1. The prescribed dose was 50 Gy in 25 fractions. A respiratory gating system, AZ-733VI (Anzai, Tokyo, Japan), was employed with an optional laser displacement sensor and another optional visual feedback device, ABLE (Anzai, Tokyo, Japan) [12]. A CBCT-equipped linac, Elekta Axesse (Elekta AB, Stockholm, Sweden), was also used. This study was approved by the institutional review board of Tokyo Medical University (approval ID: T2023-0136). Written informed consent was obtained from all 10 patients.

**Table 1:** Patient demographics. CTV: clinical target volume; PTV: planning target volume

Patient	Age	CTV volume (cm^3^)	PTV volume (cm^3^)	Heart volume (cm^3^)
A	64	518.1	864.1	475.7
B	70	363.6	621.4	393.2
C	49	63.3	196.9	463.3
D	32	396.7	654.3	539.8
E	51	419.6	739.0	541.9
F	52	561.6	1,026.6	590.2
G	34	371.9	776.3	556.5
H	44	414.2	692.2	371.4
I	44	399.7	666.3	583.7
J	48	455.4	761.3	385.5

**Figure 1:**
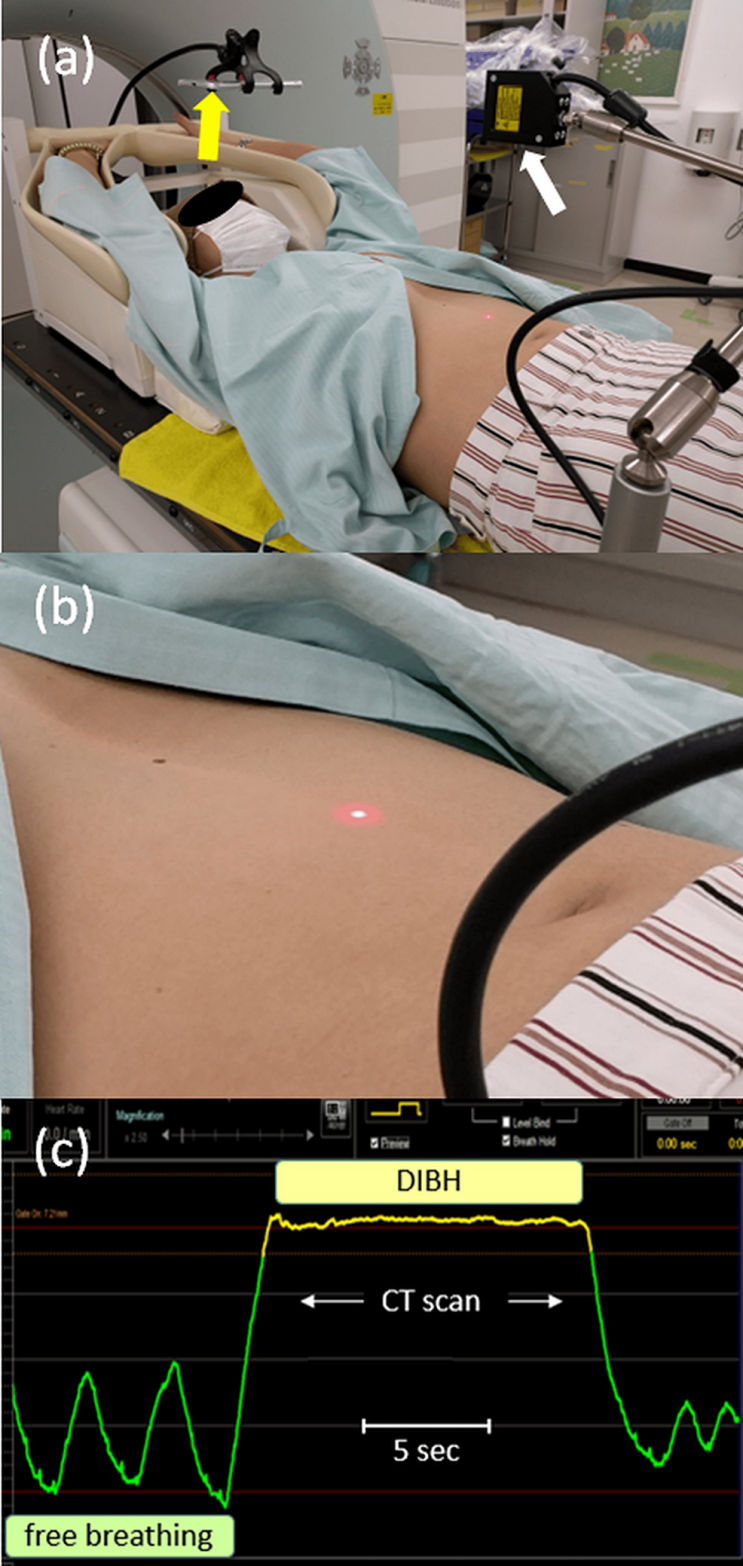
Photographs showing our planning CT scan procedure in deep inspiration breath-hold (DIBH). (a) A laser displacement sensor (white arrow) is attached to the patient couch through an articulated arm, and a compact display (yellow arrow) is placed above the patient’s head for visual feedback of the measured distance to the abdomen during the breath-hold period. (b) A narrow laser beam is projected perpendicular to the patient’s abdomen, visually midway between the xiphoid process and the umbilicus with an aimed laser-abdomen distance of 120 mm. (c) The reflected light is detected on a one-dimensional array sensor, where the detected signal position indicates the distance to the abdomen. Consequently, the free-breathing and the DIBH periods are differentiated by the laser displacement sensor. The planning CT scan was performed during the DIBH period of approximately 10 seconds.

Figures 1a-1c depict our planning CT scan procedure in DIBH. A laser displacement sensor (white arrow) was attached to the patient couch through an articulated arm, and a compact display (yellow arrow) was placed above the patient’s head for visual feedback of the measured distance to the abdomen during the breath-hold period (Figure 1a). A narrow laser beam was projected perpendicular to the patient’s abdomen, visually midway between the xiphoid process and the umbilicus with an aimed laser-abdomen distance of 120 mm (Figure 1b). The reflected light was detected on a one-dimensional array sensor, where the detected signal position indicated the distance to the abdomen (Figure 1c).

**Figure 2:**
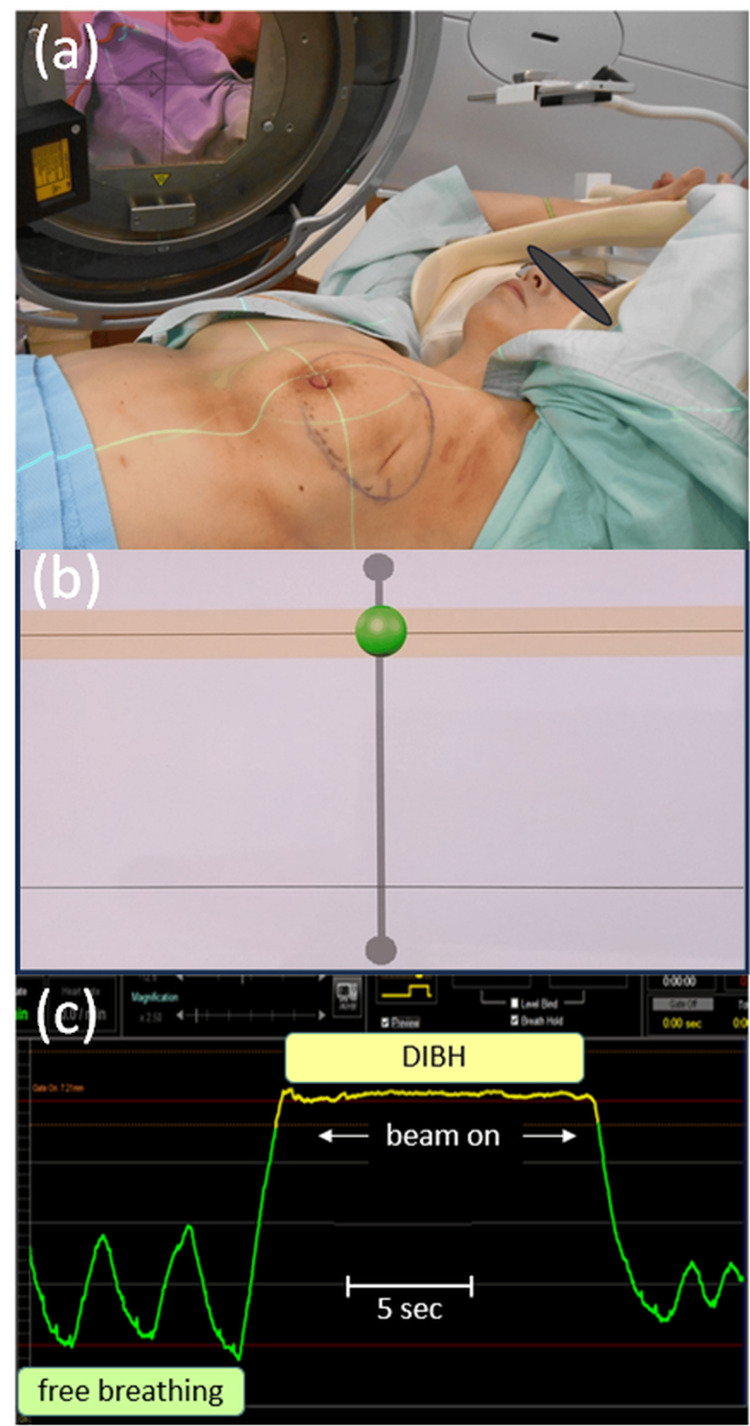
Photographs demonstrating our treatment workflow in deep inspiration breath-hold (DIBH). (a) The laser sensor and the compact display are placed in the same way as shown in Figure 1 to reproduce the measurement. (b) A display for the visual feedback is shown, where the green circle indicates the current distance to the abdomen, whereas the pink region shows an aimed range of the green circle positions. (c) A respiratory curve is shown, which is nearly identical to that shown in Figure 1c, where the treatment beam was delivered during the period of DIBH for about 10 seconds. To complete a tangential breast treatment session, the breath-hold was repeated four times.

Figures 2a-2c demonstrate the initial step of our treatment workflow in DIBH. The laser sensor and the compact display were placed in the same way as shown in Figure 1 to reproduce the measurement (Figure 2a). On a display for the visual feedback, the green circle indicated the current distance to the abdomen while the pink region showed an aimed range of the green circle positions (Figure 2b). Figure 2c shows a respiratory curve that is nearly identical to that shown in Figure 1c. Before the treatment, a half-arc CBCT image with a 200° gantry rotation was acquired during four repeated DIBHs, each spanning 50° with a 10-second breath-hold followed by five-second free breathing. The resulting CBCT image was used for the tumor registration on each day.

In this retrospective study, the change in the chest wall to the heart distance relative to the plan was calculated similarly as Benkhaled et al. reported previously [10]. We employed the Elekta X-ray Volume Imaging (XVI) dual registration [13] that allowed us to make corrections based on the tumor registration and the critical structure registration, where interpolation may be further performed between the two registration results by a clinical decision. In our study, we only conducted the two registrations with no interpolation. For simplicity, a more detailed operation of the dual registration is described in the Results section with the aid of figures. The interfractional changes among 25 fractions were depicted as a histogram and a box plot for each of the 10 patients in each coordinate axis. In addition, the median with the 95% confidence interval (CI) and the IQR for all 250 fractions were calculated to assess the reproducibility of our DIBH technique.

To assess the potential dosimetric impact of reduced distances from the chest wall to the heart relative to each plan, the MHD and the heart V25Gy were recalculated for virtual plans where the heart was displaced toward the chest wall by the largest amount measured among the 25 fractions in each direction for each patient. This may be an overestimate but is considered appropriate as a worst-case scenario. For dose calculation in the virtual plan, the mean electron density of the heart was calculated and assigned as a bulk electron density within the displaced heart volume.

## Results

A total of 250 CBCT images from the 10 patients were individually analyzed in reference to each of the planning CT images. The median age was 49 years, and the median clinical target volume (CTV), planning target volume (PTV), and heart volume were 407.0 cm³, 715.6 cm³, and 507.7 cm³, respectively.

**Figure 3:**
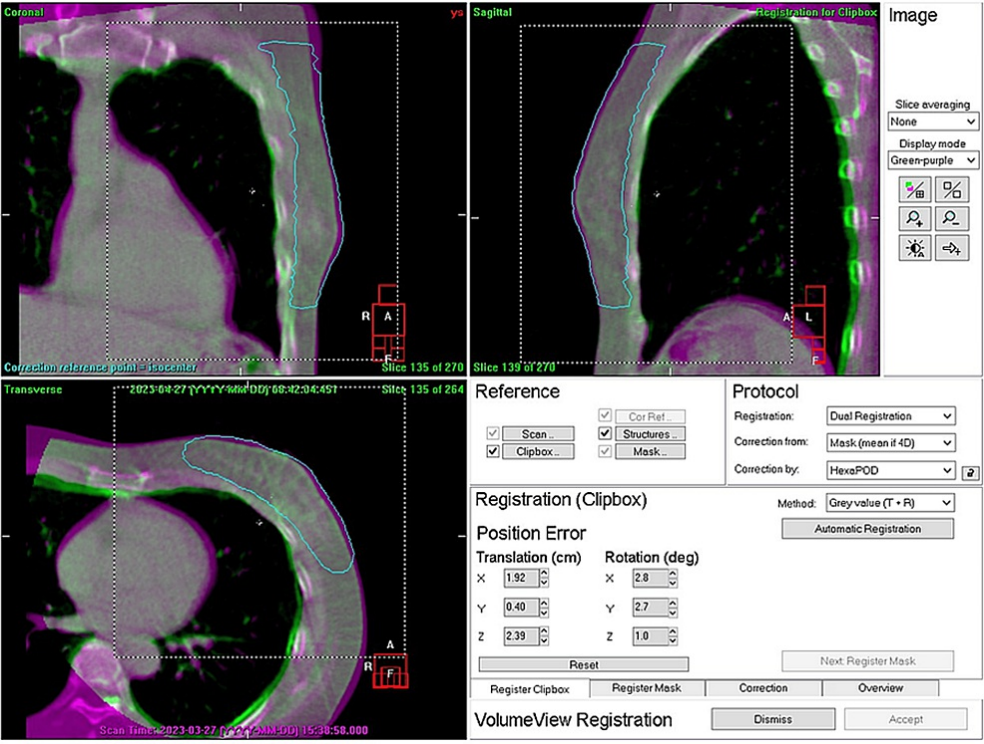
First calculation step of the chest wall to the heart distance relative to the plan. Before the treatment, a half-arc cone-beam CT (CBCT) image with a 200° gantry rotation was acquired during four repeated deep inspiration breath-holds, each spanning 50° with a 10-second breath-hold followed by five-second free breathing. Chest wall registration between the planning CT and the CBCT images was conducted using gray value matching inside a dotted white clip box. Subsequently, a manual adjustment was made as needed to match the inner chest walls, ensuring no extra doses to healthy lung tissues. The coordinate system of the Elekta linac is based on the IEC 61217 fixed coordinate system except for rotation.

Figure 3 shows the first calculation step of the chest wall to the heart distance relative to the plan. Chest wall registration between the planning CT and the CBCT images was conducted using gray value matching inside a dotted white clip box. Subsequently, a manual adjustment was made as needed to match the inner chest walls between the planning CT and the CBCT, thereby ensuring no extra doses to normal lung tissues. The coordinate system of the Elekta linac is based on the IEC 61217 fixed coordinate system except for rotation [14].

**Figure 4:**
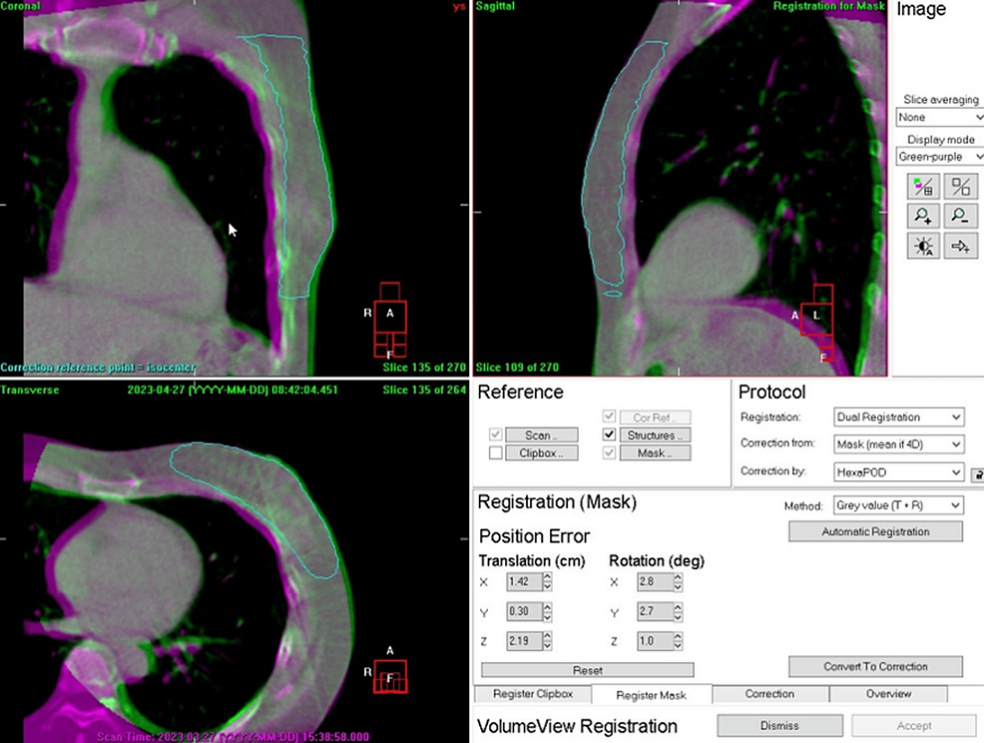
Second calculation step. Heart registration between the planning CT and cone-beam CT was performed by gray value matching inside a mask region corresponding to the heart structure defined in the plan. A subsequent manual adjustment was always conducted to match the heart surfaces proximal to the chest walls, as shown in the figure, taking into account the heart surface that may receive the highest dose.

Figure 4 indicates the second calculation step. Heart registration between the planning CT and the CBCT images was performed by gray value matching inside a mask region corresponding to the heart structure defined in the plan. A subsequent manual adjustment was always conducted to match the heart surfaces proximal to the chest walls between the planning CT and the CBCT so that the distance to the chest wall was measured from the heart surface proximal to the chest wall, which may receive the highest dose.

**Figure 5:**
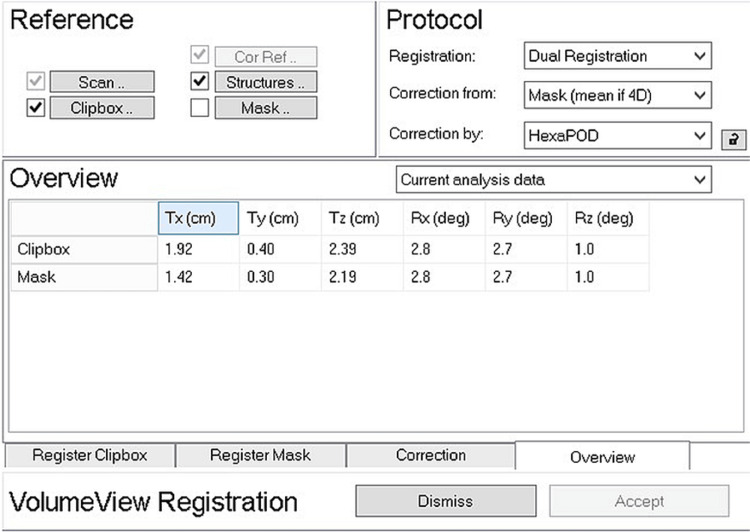
The last step. Subtraction of the heart registration result (Mask) from the chest wall registration result (Clipbox) led to a change in the chest wall to the heart distance relative to the plan. This figure shows that the changes in the distance from the chest wall to the heart relative to the plan were 0.5 cm, 0.1 cm, and 0.2 cm in X (lateral), Y (superior-inferior), and Z (anterior-posterior) directions, respectively. It is noted that the positive sign in each value indicates a larger distance relative to each plan, whereas the negative sign indicates a shorter distance. Tx: translation toward X; Ty: translation toward Y; Tz: translation toward Z; Rx: rotation around X; Ry: rotation around Y; Rz: rotation around Z

Figure 5 shows the last step. Subtraction of the heart registration result from the chest wall registration result led to the change in the chest wall to the heart distance relative to the plan. This example figure shows that the changes in the distance relative to the plan were 0.5 cm, 0.1 cm, and 0.2 cm in the X (lateral), Y (superior-inferior), and Z (anterior-posterior) directions, respectively. Of note, the positive sign in each value indicates a larger distance relative to the plan, whereas the negative sign indicates a shorter distance.

**Figure 6:**
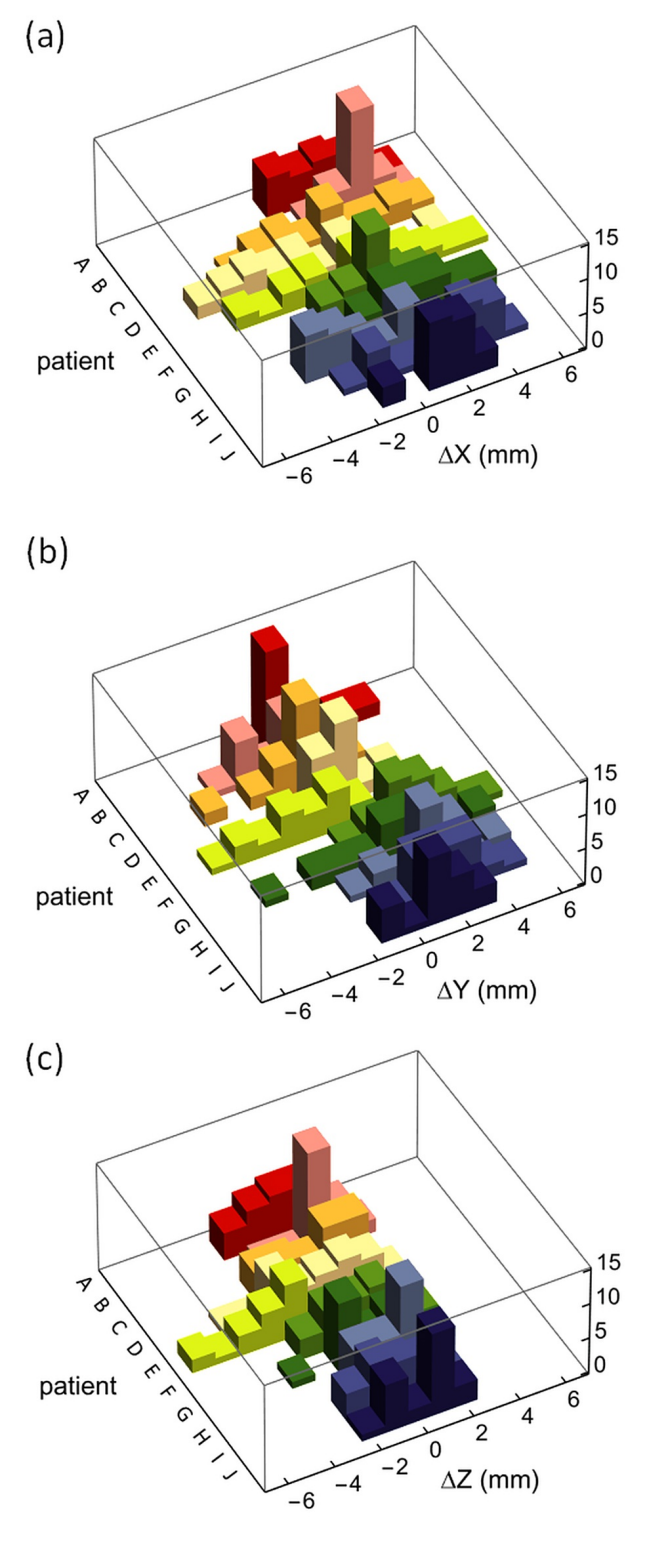
Histograms of daily changes in the distance from the chest wall to the heart relative to each plan for the 10 patients. (a) The relative change ΔX in the lateral direction, (b) ΔY in the superior-inferior direction, and (c) ΔZ in the anterior-posterior direction, where each positive change on each day indicates a larger distance from the chest wall to the heart relative to each plan, while a negative change indicates a shorter distance relative to each plan in each direction.

**Figure 7:**
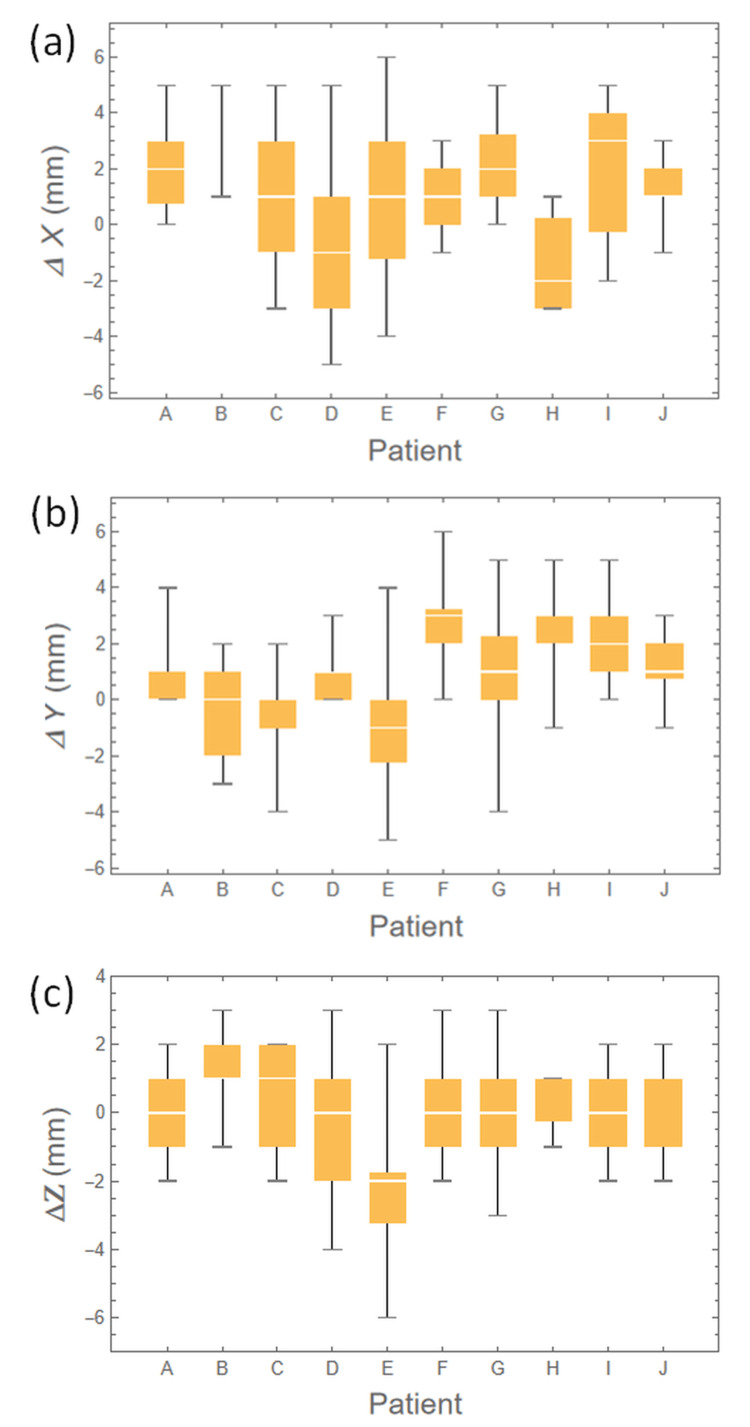
Box plots of the data shown in Figure 6. The medians of the interfractional changes in the chest wall to the heart distance in each case ranged from -2 mm to 3 mm, -1 mm to 3 mm, and -2 mm to 1 mm, in the lateral (X), superior-inferior (Y), and anterior-posterior (Z) directions, respectively. For all 10 cases, the medians were 1 mm in X, 1 mm in Y, and 0 mm in Z directions, whereas the IQRs were 4 mm in X, 2 mm in Y, and 2 mm in Z directions.

Figure 6 and Figure 7 depict histograms and box plots of interfractional changes in the distance from the chest wall to the heart relative to each plan for the 10 patients in X, Y, and Z directions, where a positive change indicates a distance larger than the plan, whereas a negative change indicates a shorter distance. In Figure 7a, the box plot for patient B in the X direction does not indicate quartiles as the 25th, 50th, and 75th quartiles are identical due to their very narrow distribution, as shown in Figure 6a. The medians of the interfractional changes in the chest wall to the heart distance in each of the 10 patients ranged from -2 mm to 3 mm, -1 mm to 3 mm, and -2 mm to 1 mm, in the X, Y, and Z directions, respectively. For all 10 cases, the medians were 1 mm (95% CI = 0.72 to 1.28 mm) in X, 1 mm (95% CI = 0.76 to 1.24 mm) in Y, and 0 mm (95% CI = -0.20 to 0.20 mm) in Z directions, whereas the IQRs were 4 mm in X, 2 mm in Y, and 2 mm in Z directions.

Each daily treatment session after the second fraction took approximately 10 minutes from room-in to room-out. The first fraction required an additional one minute to reconfirm the breath-hold procedure before treatment. Table 2 demonstrates that the MHD and the heart V25Gy after the virtual heart displacement for each of the 10 cases were no more than 2.62 Gy and 0.02%, respectively.

**Table 2:** Comparisons of the mean heart dose and the heart V25Gy between clinical plans and virtual plans. The heart in each virtual plan was displaced toward the chest wall by the largest amount measured in each direction for each patient. In other words, the largest ΔX, ΔY, and ΔZ with negative signs among all fractions were considered virtual maximums of unfavorable displacements for each patient. V25Gy: the percentage volume receiving 25 Gy or more

Patient	Mean heart dose (Gy)		Heart V25Gy (%)	
	Clinical plan	Virtual plan	Clinical plan	Virtual plan
A	1.27	1.36	0	0
B	1.3	1.39	0	0
C	0.62	0.74	0	0
D	1.58	2.44	0	0.02
E	1.52	2.62	0	0.02
F	1.75	2.04	0	0.01
G	2.02	2.58	0	0.01
H	1.21	1.37	0	0
I	1.13	1.27	0	0
J	0.85	0.91	0	0

## Discussion

Dual registration using the daily CBCT and planning CT images showed that the medians of the changes in the chest wall to the heart distance among 25 fractions in each of the 10 cases ranged from -2 mm to 3 mm, -1 mm to 3 mm, and -2 mm to 1 mm in the X, Y, and Z directions, respectively. In addition, the reproducibility of the chest wall to the heart distance in our study is substantially better than previously reported two different studies where visual feedback was not employed [9,10]. In particular, our measured IQRs were 4 mm in X, 2 mm in Y, and 2 mm in Z directions for all 250 fractions, whereas the largest distance reductions relative to the plans were 5 mm toward the left, 5 mm toward the superior, and 6 mm toward the anterior directions. Our IQRs were two to three times smaller than those reported in a previous study without visual feedback [10]. More specifically, their IQRs were 8.0 mm in X, 4.8 mm in Y, and 6.4 mm in Z directions for 225 fractions (15 cases each with 15 fractions), while the largest distance reductions were 21 mm toward the left, 9 mm toward the superior, and 18 mm toward the anterior directions. In addition, large inter-individual variations in the distance were also reported [10], which was not observed in our cases presumably due to the visual feedback. We hypothesized that the visual feedback may increase the interfraction reproducibility of the chest wall to heart distance. The results of this study may support the hypothesis, suggesting a clinical advantage of visual feedback in DIBH for left-sided breast cancer radiotherapy.

It was also reported that the entire DIBH treatment time using an optical surface scanning unit for breast cancer required 45 minutes for the first fraction and 30 minutes for the remaining fractions [15]. Our DIBH procedure with the Anzai laser sensor with visual feedback is considered highly efficient, which takes approximately 10 minutes from room-in to room-out, thereby not reducing the daily number of patients. Our treatment duration is also shorter than that shown in a report including 10 UK hospitals employing vDIBH for breast cancer radiotherapy, where the institutional medians ranged from 13 to 24 minutes and the inter-institutional median was 21 minutes [7].

As shown in Table 2, the calculated MHD and the heart V25Gy in the virtual plan for each patient were all below 3.3 Gy and 5%. As these criteria reportedly reflect a very low risk of radiation-induced cardiac death at 10 years [3], our DIBH radiotherapy may provide similar favorable outcomes to the patients studied.

Limitations of this study include the sample size that is too small to generalize the results. In addition, we did not compare the same patient population with and without visual feedback.

## Conclusions

We have studied the reproducibility of the chest wall to heart distance using our DIBH solution with visual feedback. Our findings are that the DIBH with visual feedback achieved better distance reproducibility, which suggests less dose to the heart. Our DIBH solution required approximately 10 minutes from room-in to room-out, thereby not reducing the number of patients to be treated per day. To our knowledge, this is the first paper reporting the chest wall to heart distance reproducibility in DIBH with visual feedback.
